# Laypeople perception and interpretation of simulated life-threatening bleeding: a controlled experimental study

**DOI:** 10.1186/s12873-021-00496-2

**Published:** 2021-09-04

**Authors:** Erik Prytz, Rachel Phillips, Susanna Lönnqvist, Marc Friberg, Carl-Oscar Jonson

**Affiliations:** 1 Department of Computer and Information Science, Linköping University, Linköping, Sweden; 2 Center for Disaster Medicine and Traumatology, and Department of Biomedical and Clinical Sciences, Linköping University, Linköping, Sweden; 3 Department of Psychology, Old Dominion University, Norfolk, Virginia USA

**Keywords:** Blood loss estimation, Bleeding control, Training, Laypeople, Immediate responder

## Abstract

**Introduction:**

First aid performed by immediate responders can be the difference between life and death in the case of trauma with massive bleeding. To develop effective training programs to teach bleeding control to laypersons, it is important to be aware of beliefs and misconceptions people hold on bleeding and severity of bleeding situations.

**Method:**

A controlled study was conducted in which 175 American college students viewed 78 video clips of simulated bleeding injuries. The volume of blood present (between 0 and 1900 ml), rate of blood flow, and victim gender were systematically varied within participants. Participants were asked to rate injury severity, indicate the appropriate first aid action, and estimate the amount of time until death for the victim.

**Results:**

Though the Stop the Bleed® campaign recommends training laypeople to treat 165 ml of blood loss as life threatening, participants largely rated this volume of blood loss as minimal, mild, or moderate and estimated that the victim had just under one hour to live. Increased blood loss was associated with increased recommendations to use a tourniquet. However, in the 1900 ml conditions, participants still estimated that victims had around 22 minutes to live and approximately 15% recommended direct pressure as the intervention. Severity ratings and recommendations to use a tourniquet were also higher for the male victim than the female victim.

**Conclusions:**

Injury classification, intervention selection, and time to death-estimations revealed that training interventions should connect classifications of blood loss to appropriate action and focus on perceptions of how much time one has to respond to a bleeding. The study also revealed a gender related bias in terms of injury classification and first aid recommendations. Bleeding control training programs can be designed to address identified biases and misconceptions while building on existing knowledge and commonly used terminology.

**Supplementary Information:**

The online version contains supplementary material available at 10.1186/s12873-021-00496-2.

## Introduction

Injuries can happen at any time and place. When they do, interventions from people present at the scene of the accident, so-called immediate responders, can be the difference between life and death [1]. Immediate responders may have little medical knowledge or expertise. First aid training is not designed to turn laypeople into medical experts, but rather to provide laypeople with basic skills and knowledge to correctly implement potentially life-saving interventions until experts can arrive. Thus, designing effective first aid training programs is important. To facilitate effective program design, it is necessary to determine not only what information and skills need to be acquired but also the way in which these may interact with the knowledge and beliefs that laypeople already hold.

Lay knowledge is knowledge acquired over time in everyday life through personal experiences and interactions [2]. It contrasts with expert knowledge, which is characterized by specialized information, enhanced knowledge organization, and facilitated cognitive functioning in the field of expertise [3]. Although they lack training, laypeople also possess naïve theories formulated on an abstract, general level starting early in life [2]. According to Wellman and Gelman [4] there are three core theories formed early in the lifespan that impact later learning: naïve physics, naïve psychology, and naïve biology. As everyone has experienced illness at some point in their lives, medical laypersons possess lay knowledge and naïve theories about illness and well-being [5]. Failing to account for naïve theories and misconceptions held by laypeople may hamper the design of effective educational interventions [6].

In the last five years, many educational interventions have been created related to the control of massive hemorrhage due to trauma [7]. Blood loss due to traumatic injury is a major cause of death globally [8, 9]. Intervention by laypeople is often necessary as a massive traumatic hemorrhage can lead to death of the victim in minutes [10], which may be too little time for professional first responders to arrive [1].

The Stop the Bleed® campaign aims to teach the general population to recognize and stop life-threatening hemorrhage [11–14]. However, it is unclear how the lay knowledge and naïve theories medical laypeople have concerning blood loss affects these educational interventions. For instance, one recommendation from the Stop the Bleed Education Consortium is that laypeople without prior medical knowledge should be taught very simple guidelines for how to recognize a life-threatening blood loss. One of these guidelines is that if the volume exceeds half a soda can, which is about 165 ml, it should be treated as life-threatening [11]. Knowing how laypeople perceive blood loss of different volumes could inform the educational design of interventions aiming to teach this guideline.

A further problem in teaching bleeding control is that blood loss estimations by both laypersons and medical professionals are largely inaccurate and underestimated at high volumes [15–18]. Thus, even if a layperson knows that 165 ml of blood loss should be treated as life-threatening, they may misperceive the amount of blood present or may misclassify the severity of the injury and respond inappropriately. We previously examined the ability of laypeople to correctly estimate the amount of blood lost and classify injuries dichotomously as life-threatening or not [18]. However, there is still a need to examine how medical laypeople perceive blood loss in terms of severity and necessary first aid interventions. From an ontological perspective it makes sense to determine how medical laypeople classify blood loss using a standardized scale. The distribution of the words used on the severity scale given different volumes of blood loss could be used to design training interventions and as a way to facilitate communications between medical laypeople and medical professionals when discussing blood loss. It could, for example, be useful as a guide for what words to use when teaching how to estimate blood loss or to be aware of what an immediate responder may mean when they use a certain word.

In addition to injury classification tendencies, it would also be useful to examine naïve theories regarding understanding the urgency of the situation. This could be done by looking at estimated time to death of a bleeding victim given different volumes of blood loss. These estimations could be relevant in two ways: First, estimated time to death could serve as an indicator of perceived urgency outside of structured classifications; Second, estimated time to death may impact the likelihood that an immediate responder would act while waiting for first responders to arrive. If perceived injury severity and temporal urgency impact first aid recommendations or the likelihood of intervention, these would need to be addressed with more targeted training.

Finally, there is evidence of potential gender related biases in medical care [19–21]. Though the effect of victim gender had not previously been examined in the context of blood loss estimation [22], Phillips et al. [18] found that blood loss was underestimated more for the female victim than the male victim, and the blood loss was also less likely to be classified as life threatening. It is possible that naïve gender-related biological or psychological theories may influence various aspects of the first-aid decision making process. In this case, victim gender may impact severity estimations, estimated time to death, and recommended first aid actions.

Thus, the current study sought to examine the relationship between victim blood loss and estimations of time to death, injury severity classification, and recommended first aid actions made by medical laypeople. To accomplish this, participants were asked to view a series of short video clips of a patient with a simulated bleeding. The participants were asked to provide time to death estimates, classify the injury severity using the verbal descriptors from the SPOT GRADE bleeding severity scale [23], and indicate which first aid action would be most appropriate. We also examined the effect of victim gender on estimations and recommendations made by medical laypeople, as half of the videos featured a male victim while the other half featured a female victim.

## Method

### Study participants

This study is based on data collected in the controlled experiment reported in Phillips et al. [18] and is described in more detail there.

A total of 175 participants were recruited from the undergraduate population of a university in the south eastern part of the United States through an online participation system. Of these, 50 had prior experience working in healthcare or stop the bleed-training and were therefore excluded from the analyses. The remaining 125 participants (21 male, 104 female) had an age range of 18–59 years (*M* = 20.1 *SD* = 4.9). The participants received research credits toward extra credit or class required research experience credit. All APA (The American Psychological Association) ethical guidelines were followed.

### Data collection

Participants viewed 78 5-secods video clips of a patient actor bleeding from a simulated wound on the upper thigh. To eliminate the potential of confounding factors such as location, wound appearance, or setting, the patient actors were presented in identical seated positions against a plain, white wall on plastic flooring in each video. They were dressed in identical blue, short-sleeved hydrophobic scrubs and bleeding from their right inner thigh. The bleeding was created using porcine blood pumped through a plastic tube, which was hidden from view and terminated at the edge of the scrub shorts. Victim gender (male or female), blood loss amount (13 volumes in ml: 0, 50, 100, 150, 200, 300, 400, 500, 700, 900, 1100, 1500, and 1900), and flow rate (80, 200, and 400 ml per minute) were systematically varied in the videos. Though the videos were filmed from two viewing perspectives (front and top-down), perspective was not found to impact blood loss estimations [18] and was dropped as a variable in this analysis. Flow rate was also not included as a variable outside of the creation of the videos as there was a concern that participants would be unable to discriminate between flow rates in such a short amount of time.

The experiment was conducted in a lab with four computers. The participants read and signed an informed consent form on paper and were then directed to use a computer to perform the experiment. The computers used the System for Acquiring Blood Loss Estimates (SABLE), an online tool developed to standardize the data collection procedure. SABLE first showed instructions to the participants, informing them that they were to view short videos of simulated bleeding, that their task was to determine if the bleeding shown was life-threatening or not, and to answer questions related to the amount of blood loss and severity of the bleeding. Participants clicked start when they were ready to begin.

Participants viewed video clips for 5 seconds each. They were asked to classify the bleeding as life threatening or not life threatening. After the video disappeared, participants were asked to estimate blood loss, indicate bleeding severity according to the SPOT GRADE scale [23] (None; Minimal; Mild; Moderate; Severe – not immediately life-threatening; or Extreme– Immediately life-threatening), choose an intervention (No action; Stop the blood flow by pressing hard on the wound using your hands and, if possible, an absorbent material; or Stop the blood flow by putting something (like a tourniquet or strap) above the wound and tightening until the bleeding stops), and to estimate the time until death in minutes. The participants could choose to give their estimate in fluid ounces or milliliter by typing into a corresponding box. This free choice of units was provided because some participants might be unfamiliar with milliliters. All estimates were converted to milliliters for the analyses.

The 78 videos were shown in a random order for each participant. Participants were presented with a demographic questionnaire asking for gender, age, and prior experience with healthcare or stop the bleed-training at the end of the experiment. After the demographic questionnaire, participants were provided a debrief onscreen and a short video of the female actor holding a sign saying “Thank you” to help offset any negative emotional reactions from the experiment and to reinforce the understanding that the videos were simulations. Finally, participants were thanked for their participation and debriefed by the experimenter prior to leaving the lab.

### Data analysis

Significance tests were conducted using Student’s t-test with *p* ≤ 0.05 considered significant. Descriptions of distribution of injury severity classification and appropriate first aid action are provided. Simple regression analyses were performed on estimated time to death and blood loss volume in ml. The analyses were done in SPSS (Statistical Product and Service Solutions) and graphs were constructed in Microsoft Excel.

## Results

### Injury severity classification

**Fig. 1:**
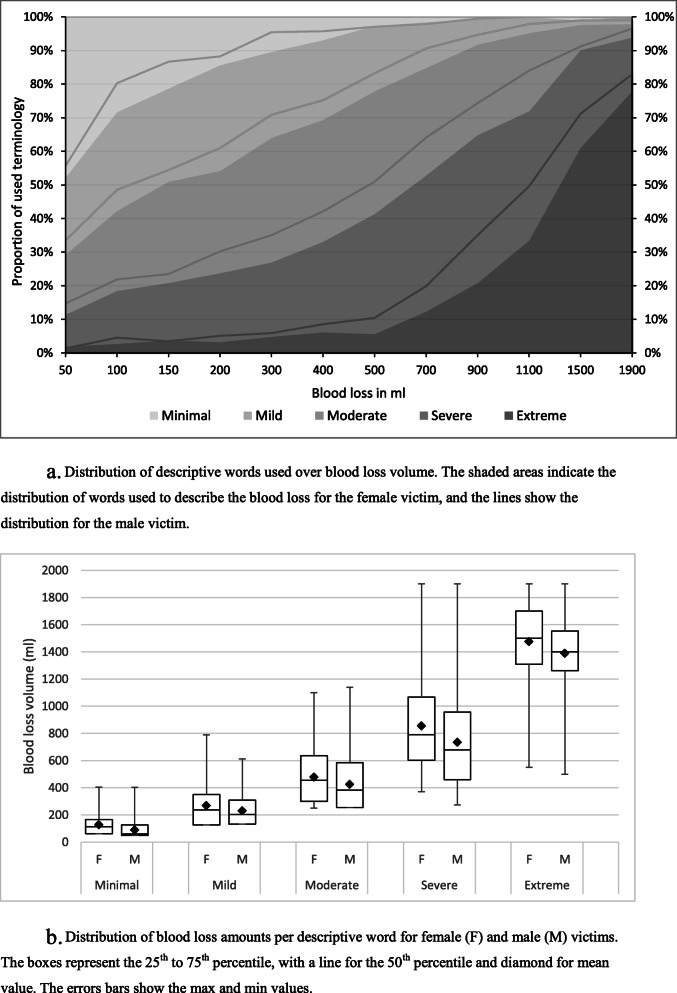
**A**. Distribution of descriptive words used over blood loss volume. The shaded areas indicate the distribution of words used to describe the blood loss for the female victim, and the lines show the distribution for the male victim. **B**. Distribution of blood loss amounts per descriptive word for female (F) and male (M) victims. The boxes represent the 25th to 75th percentile, with a line for the 50th percentile and diamond for mean value. The errors bars show the max and min values

Figure 1A shows the distribution of bleeding severity words as a function of the blood loss shown in the videos. As indicated by the darker colors in Fig. 1A, classification of blood loss as severe or extreme was more common at higher levels of blood loss. At 150 ml of blood loss, the closest volume corresponding to half a soda can, the severity was predominantly classified as minimal (female victim 21.3%; male 13.3%), mild (female 27.7%; male 32.3%), or moderate (female 30.1%; male 30.9%). Figure 1A also shows that the male injuries were consistently classified as more severe than female injuries at the same level of blood loss, as the lines (male victim) are always higher than the shaded areas (female victim). This occurred even at the largest volumes of blood loss (1500 ml and 1900 ml).

Figure 1B shows the mean blood loss associated with each descriptive word for each gender. Paired t-tests showed that the mean blood loss value was significantly lower for the male patient for all descriptive words (all *p* < .001 or below) except for in the case of “None” where there was no difference, *t*(123) = 0.369, *p* = .713. In other words, if bleeding was present, a larger blood loss was required on average before the participants applied the same descriptive word for the female victim as for the male victim.

### Minutes to death

**Fig. 2:**
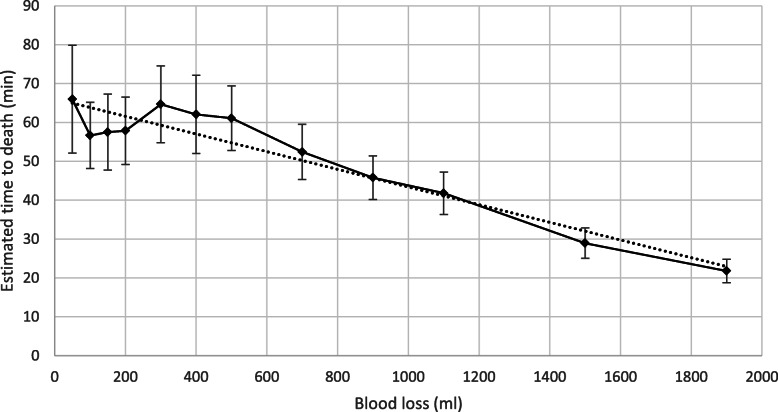
Mean estimate of time to death over blood loss amount. Error bars show 95% confidence intervals. The dotted line indicates the fitted linear trend line

The participants were asked to estimate how long, in minutes, they thought the patient was from death given the bleeding and blood loss they had just seen in the video if first aid was not administered. As can be seen in Fig. 2, estimation of time to death was about 1 h (*M* = 57.4, *SE* = 5.0) for a blood loss of 150 ml. As might be expected, estimations of minutes to death decreased with blood loss volume and the lowest estimations occurred at the highest volume of blood loss with estimations around 22 min (*M* = 21.8, *SE* = 1.5) for 1900 ml. Figure 2 shows the mean estimate with 95% confidence intervals. Videos showing no blood loss were excluded from this analysis. A fitted trend line (dotted), R^2^ = .908. is shown, with the function given by Y = − 0.023x + 66.15. This shows that the estimate for time to death given a low blood loss (50 ml) starts at just over an hour (specifically a mean of 66 min, 95% CI [52, 80]), and the estimate then decreases by one minute per ca 50 ml (specifically 44.05 ml) or ca 1.5 fluid ounces (specifically 1.49 fl. oz.).

### Appropriate action

**Fig. 3:**
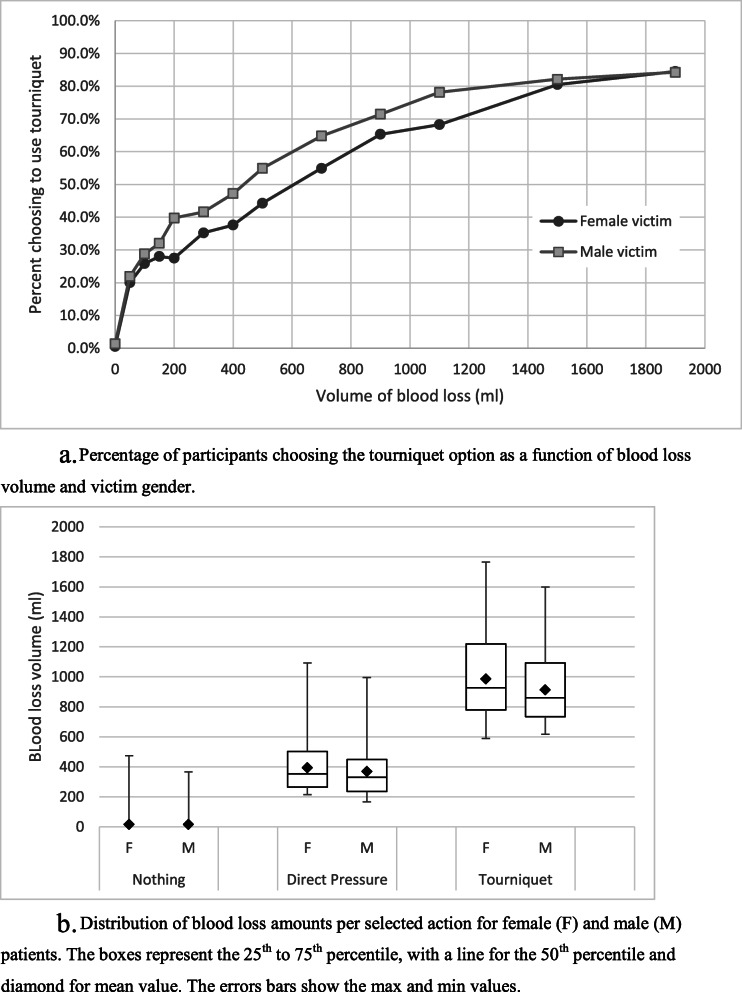
**A**. Percentage of participants choosing the tourniquet option as a function of blood loss volume and victim gender. **B**. Distribution of blood loss amounts per selected action for female (F) and male (M) patients. The boxes represent the 25th to 75th percentile, with a line for the 50th percentile and diamond for mean value. The errors bars show the max and min values

Participants were asked to choose which action would be appropriate for the blood loss depicted in the videos: no action, direct pressure, or tourniquet/strap above the wound. As can be seen in Fig. 3A, increased blood loss was associated with increased recommendations to use a tourniquet, though 15.3% still recommended using only direct pressure even at the largest volume of blood loss. At 150 ml of blood loss a little less than a third of the respondents selected the tourniquet option (28% for the female victim; 32% for the male). Tourniquets were selected more often for the male victim than the female victim for the conditions depicting 200–1100 ml of blood loss. At 1500 and 1900 ml, tourniquet selection was similar between genders.

Figure 3B shows the mean blood loss associated with each action selection. The mean volume of blood loss was higher for the female victim for the direct pressure, *t*(119) = 2.904, *p* = .004, and tourniquet actions, *t*(123) = 6.221, *p* < .001. This indicates that a higher blood loss was required, on average, before the participants chose the direct pressure or tourniquet alternatives for the female victim as compared to the male victim.

**Fig. 4:**
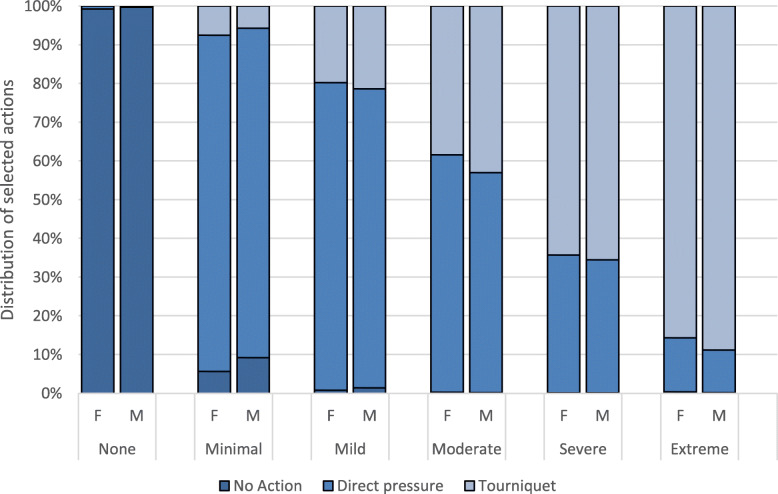
Distribution of the selected action over descriptive word for female (F) and male (M) patients

Further, Fig. 4 shows the relationship between the selected action and the words used to describe the injury. As shown previously in Figures 1AB and 3AB, the choice of descriptive word varied between the genders and blood loss volume and so did the recommended action. Figure 4 shows that there is a small difference in the chosen action between genders across the different severity levels. This seems to indicate that the higher frequency of tourniquet use for male victims is driven more by the perception of injury severity than by a bias in action selection. Put plainly, participants were likely to select the same action (i.e., direct pressure or tourniquet) given a perceived severity (such as mild or moderate). However, they were more likely to classify male injuries as more severe than female injuries given the same amount of actual blood loss, and subsequently also recommend either direct pressure or tourniquet use more for the male victim.

## Discussion

To develop successful interventions for medical laypersons responding to bleeding injuries, it is necessary to first understand their preexisting lay knowledge. Misconceptions can hinder learning, as they are difficult to change [24, 25]. In the case of first aid for massive bleeding, training non-professional immediate responders to properly identify a life-threatening bleeding and the acuity of the situation is important to increase potential survivability of trauma injuries. Thus, the current study sought to examine the relationship between victim blood loss and estimations of time to death, injury severity classification, and recommended first aid actions made by potential immediate responders to investigate what conceptions people hold about bleeding injuries.

One of the educational guidelines from the Stop the Bleed Education Consortium is that laypeople should be taught that half a soda can, or about 165 ml, of blood loss is life threatening [11]. This guideline recommendation is based on the idea that a person who has lost approximately 165 ml of blood and is continuing to bleed could reach truly life-threatening volumes of blood loss before first responders can arrive. Therefore, immediate responders should act early, before the blood loss becomes life-threatening. The current study revealed that most participants categorized 150 ml of blood loss as minimal, mild, or moderate. This finding indicates that educators may need to emphasize the time sensitive nature of hemorrhage control to convince learners to use “half a soda can” as a decision heuristic for life-threatening bleeding. Further, fewer than a third of the participants opted to use a tourniquet for this level of blood loss. Even at very high levels of blood loss, there were still respondents who opted for direct pressure rather than tourniquets and classified the injury as not immediately life threatening.

The participants also estimated that a person with a 150 ml blood loss would take, on average, almost an hour to die if no first aid was administered. Though the average estimated time to death did decrease as the volume of blood loss increased, participants still estimated almost 22 minutes until death for 1900 ml of blood loss. This suggests that interventions need to focus on shifting not only the classifications of blood loss and when particular interventions would be best, but also perceptions of how much time one has to effectively respond to a bleeding injury. Training interventions can be designed to address identified misconceptions while building on existing knowledge and commonly used terminology. In training for massive bleeding events and to support restructuring of concepts about bleeding, simulation training where students may observe the circulatory system mechanics and the physiology of bleeding could potentially support the development of more accurate mental models. Such programs would likely result in increased training effectiveness as they would focus more on skill development in targeted areas and would be susceptible to fewer misinterpretations by students.

The current study also revealed a gender related bias for medical laypeople in terms of injury classification and first aid recommendations. Specifically, the same blood loss volume was classified as being less severe for women than for men. Additionally, greater blood loss was required for the female victim before the participants chose the direct pressure or tourniquet option, as compared to the male victim. However, there were relatively small differences between genders for the recommended actions at each injury severity classification (mild, moderate, etc.). This suggests that the differences in tourniquet recommendations between genders may stem primarily from biases impacting the classification of injury severity. This may be due to perceptual differences for the amount of blood present [18], the location of the injury, or misconceptions of women in acute medical distress. As reported in Perman et al. [26], there were three main themes for why women were less likely to receive CPR (cardiopulmonary resuscitation) when suffering an out of hospital cardiac arrest: sexualization of women’s bodies, perceived proneness to injury, and beliefs that women were generally healthier and more prone to exaggeration than men. The simulated bleeding injury in the current study was quite high on the thigh, participants may have felt uncomfortable looking at the area as closely for the female actor. These possibilities need to be examined in future research to determine the best way to address and reduce this bias.

Future research should also include a wider range of victim actors and settings. The current study used the same two actors in the same setting for all conditions. Although this allowed for greater experimental control, it reduces the generalizability of results. The effects of setting and victim characteristics such as age, ethnicity, BMI (body mass index), or clothing should be examined to determine additional areas to focus on when training laypeople. The setting itself is likely to be important as well, as the perceived time until help arrives is likely to influence the behavior of the immediate responders. Another area of future research is to investigate more in depth which decision heuristics untrained laypeople rely on to understand and make decisions about blood loss and trauma. For example, if the soda can metaphor serves as a useful anchor for laypeople’s decision making about life-threatening bleeding. A comparison before and after stop the bleed training could be informative in this respect.

In this study, participants were asked to choose a descriptive word for each video from a set of given words. It is important to mention that these descriptive words might not be the words laypeople actually use to describe life-threatening bleedings in real-world settings. Previous research has shown that people describe volumes in, for example, graphical (e.g., “a lot” or “big”) or emotional (“crap load”) terms [27]. As such, in educational settings, it is important to acknowledge and examine what appropriate words or terms should be used by educators when teaching hemorrhage control to laypeople in different cultures or age groups. Another aspect to consider in future studies is the effect of media. Repeated exposure to depictions of injury, its consequences, and unrealistic survival in TV-shows and video games may influence the estimation and severity perception of bleedings, similarly to how crime shows can affect people’s beliefs and expectations regarding forensic evidence (the CSI-effect) [28]. Aspects of frequency of use of such media and perceived realism could be incorporated in future studies. Finally, due to the number of stimuli present in the current experiment, participants had limited time to view each video clip. It is possible that longer viewing times would allow for more accurate classifications and associated first aid decisions. Future research should examine this possibility as it could inform training design and research methodology.

Understanding the naive theories and conceptions that medical laypeople hold could be used to inform educational priorities and facilitate conceptual change. Ultimately, the goal is to promote accurate and speedy first aid interventions. In the area of traumatic hemorrhage, this study identifies several areas in terms of injury classification, intervention selection, time to death-estimations, and gender where training could be enhanced to improve victim outcomes.

## Supplementary Information

**Additional file 1.**

## Data Availability

The datasets used during the current study are available from the corresponding author on reasonable request.

## Abbreviations

APA: The American Psychological Association
BMI: body mass index
CPR: cardiopulmonary resuscitation
SABLE: System for Acquiring Blood Loss Estimates
SPSS: Statistical Product and Service Solutions
