# Supplementary material for: Laypeople perception and interpretation of simulated life-threatening bleeding: a controlled experimental study
Source: BMC Emerg Med. 2021 Sep 4;21:100. doi: 10.1186/s12873-021-00496-2 (PMC8418730; doi:10.1186/s12873-021-00496-2)
Supplement: Supplementary file 1 — Additional file 1. [file 12873_2021_496_MOESM1_ESM.docx]

**Supplemental material to “Laypeople perception and interpretation of simulated life-threatening bleeding: A controlled experimental study”**

**Experimental Instructions**

Thank you for your participation today. The remainder of the experiment will be completed on the computer. You will be completing a study in which you are asked to view short video clips of simulated injuries after which you will estimate the amount of blood an individual is losing and indicate what the appropriate first aid action would be. We ask that you refrain from eating or drinking during the study. Additionally, please turn off all electronic devices and place them in your bag (or face down on the corner of the desk) for the duration of the study. If you require a break or have any questions, please let the experimenter know. When you are ready to begin, please click start on the screen and follow the instructions.

**Questions shown after each video**

1. How much blood had the individual in the video lost? ____
2. How would you describe the flow of blood?
   1. None
   2. Oozing
   3. Pooling/dripping
   4. Flowing
   5. Streaming
   6. Gushing
3. How would you classify the injury?
   1. None
   2. Minimal
   3. Mild
   4. Moderate
   5. Severe-not immediately life threatening
   6. Extreme-immediately life threatening
4. What is the most appropriate action for the current injury?
   1. No action
   2. Apply direct pressure
   3. Apply tourniquet
5. If left untreated, how long (in minutes) would it take the person to die? Put N/A if you do not think the injury could result in death. ____
